# The Importance of Endophenotypes to Evaluate the Relationship between Genotype and External Phenotype

**DOI:** 10.3390/ijms18020472

**Published:** 2017-02-22

**Authors:** Marinus F. W. te Pas, Ole Madsen, Mario P. L. Calus, Mari A. Smits

**Affiliations:** 1 Animal Breeding and Genomics Centre, Wageningen UR Livestock Research, 6700AH Wageningen, The Netherlands; mario.calus@wur.nl (M.P.L.C.); mari.smits@wur.nl (M.A.S.); 2 Animal Breeding and Genomics, Wageningen University, 6700AH Wageningen, The Netherlands; ole.madsen@wur.nl

**Keywords:** livestock science, genomic variation and environment, methylome, transcriptome, proteome, metabolome, phenome, integration, bioinformatics, systems biology

## Abstract

With the exception of a few Mendelian traits, almost all phenotypes (traits) in livestock science are quantitative or complex traits regulated by the expression of many genes. For most of the complex traits, differential expression of genes, rather than genomic variation in the gene coding sequences, is associated with the genotype of a trait. The expression profiles of the animal’s transcriptome, proteome and metabolome represent endophenotypes that influence/regulate the externally-observed phenotype. These expression profiles are generated by interactions between the animal’s genome and its environment that range from the cellular, up to the husbandry environment. Thus, understanding complex traits requires knowledge about not only genomic variation, but also environmental effects that affect genome expression. Gene products act together in physiological pathways and interaction networks (of pathways). Due to the lack of annotation of the functional genome and ontologies of genes, our knowledge about the various biological systems that contribute to the development of external phenotypes is sparse. Furthermore, interaction with the animals’ microbiome, especially in the gut, greatly influences the external phenotype. We conclude that a detailed understanding of complex traits requires not only understanding of variation in the genome, but also its expression at all functional levels.

## 1. The Definition of Complex Traits in Livestock Science

Although most livestock traits are found to be complex, there are a few examples where a trait is caused by a single gene. Examples of single gene traits include the susceptibility for the development of porcine stress syndrome (PSS) caused by a recessive gene mutation in the ryanodine receptor gene, double muscling (DM) in cattle caused by different mutations in the myostatin (also called *GDF-8*) gene, Polled/hornless (*interferon gamma receptor 2*), PRRSV (Porcine reproductive and respiratory syndrome virus) susceptibility (*heparin sulfate*, *sialoadhesin*, *CD163*, *CD151* and *vimentin* have all been identified as receptors, each explaining part of the biological receptor mechanism; moreover, it has been shown that disrupting this sequence of receptor actions by taking out at least one of these (*CD163*) through gene-editing makes pigs immune for PRRSV infection), *Escherichia coli* F4 susceptibility of pigs (*Mucin 4* gene), scrapie susceptibility in sheep (3 mutations in the *PrP* gene) and bovine leukocyte adhesion deficiency (BLAD) caused by a mutation in the *CD18* gene [1,2,3,4,5,6,7,8,9,10,11,12]. Typically, these mutations disrupt the proper functioning of a protein, thereby affecting a major biological pathway and/or cellular functions (receptor adhesion). Due to their effects on selected traits (e.g., high meat percentage on the carcass (*ryanodine receptor* mutation in pigs and myostatin mutations in cattle) or high milk yield (*CD18* in cattle), these mutations were indirectly targeted by selection in breeding programs. This resulted in high allele frequency of these mutations in the selected population. Sometimes, if the trait is determined by a single gene, adverse effects were noticed of a genetic variant when homozygote animals arose. The development of specific molecular tests [1,9] made it possible to breed against these negative mutations introduced by breeding, which has been done for, e.g., PSS and BLAD.

However, most production- and disease susceptibility-related traits in livestock are not regulated by a single gene. These so-called complex traits are regulated by (the expression of) multiple genes, sometimes up to hundreds of genes, that cooperate in physiological processes best described as pathways and/or as networks of genes/proteins/metabolites [13]. Therefore, the additive effects of all genes may be the most important characteristic to study for complex traits. This was already recognized a century ago and led to the development of the infinitesimal model [14]. Genetic variation in genes or variation in gene expression does not always disrupt the proper functioning of the protein itself, but leads to small differences in the pathway functions to which they belong. The sum of all of the genetic variations in genes and their regulatory elements changes the phenotype of (complex) traits. This information about complex trait phenotypes is currently used by breeding organizations to improve the economic value of traits (i.e., selecting for the phenotype with the highest economic value) [15]. For clarity: depending on the species, the trait may differ. The traits typically include (1) production level, product quality or sustainability of production; and (2) animal health, robustness and longevity [16,17]. Because many genes are involved in the biological regulation of the phenotype of a complex trait, multiple pathways and gene networks are expected to act together in regulating the phenotype of a trait. As many (sub)pathways may also be involved in the regulation of multiply phenotypes, the selection of one trait can affect the phenotype in another, as is indicated by non-zero genetic correlations between traits. Furthermore, since multiple tissues may be involved in the regulation of traits, the biological mechanisms underlying the traits may include different (signaling) pathways in different cell types and communication between cells, and different “biological levels” are involved, like gene expression, protein expression, metabolites, microbiota, etc.

The phenotypes of complex traits are not only regulated by the combined genotypes of the participating genes. Almost all phenotypes of traits are also (quantitatively) dependent on a number of external (environmental) factors, like nutrition, management, housing conditions and climate, but are also dependent on “environments” encountered by individual cells and/or tissues/organs, etc. Apparently, these external/environmental factors influence the relationship between the genotype and the external phenotype of traits. Environmental factors do not change the genotype of an animal directly, but affect the expression and functioning of sets of genes/proteins/metabolites and/or the communication between cells/tissues. It is known that the expression level of genes can be influenced by affecting the activity of the genome directly at the RNA synthesis or stability levels or via methylation of the genome (DNA methylation, histone modifications and regulatory non-coding RNAs (ncRNA); see further in the epigenomics section). Alternatively, the translation efficiency can be affected, and the activity of the proteins could be influenced via post-translational modifications of the proteins. There are many other biological mechanisms that could affect all of these processes; just to mention one important general factor: dependency on the energy status of cells. Altered protein activity may influence the flux of metabolites through pathways. Therefore, there is a wealth of molecular and biological mechanism information available that potentially can be used in breeding programs. Using this may enable regulating traits (external phenotypes) via different genes than only quantitative trait loci (QTL) genes (that have observed or estimated associations with phenotypes). All of these options together make the research of complex traits even more challenging. A recent study [18] showed the power of the integration of data from most of these biological levels in explaining different traits using 90 mouse inbred strains. To make it even more complex, recent investigations pointed to the importance of the microbiome for the metabolism and phenotype of the production and health traits of human and livestock [19,20,21,22,23]. Therefore, exploring the genotype to phenotype relationship is required to get the understanding of the nature and regulation of the complex traits in livestock science. This knowledge can be used to improve complex traits in breeding, nutrition, animal management, etc.

## 2. Phenome

The phenotype of a trait is how the trait is expressed externally: e.g., the amount of milk, the number of eggs, the amount of meat on a carcass. More complex, but still external phenotypes are quality traits, including traits like the composition of the milk (important for consumers, but also for the processing industry), meat quality (taste and health aspects for consumers) and animal health, adaptability, robustness, resilience, etc. Below, we will discuss that all livestock phenotypes are the result of biological processes at various biological levels. The profile of components active at a certain biological level and contributing to the development of the external phenotype constitute phenotypes by themselves, more specifically endogenous phenotypes, or endophenotype [24,25,26], as they are not readily visible from the outside. An endophenotype can be defined as a quantitative biological (sub)trait that is reliable (measurable) and reasonably heritable [27]. A complex trait arises from many genes, i.e., the existence of many genes with variation at the DNA or expression levels, whose individual (internal) components each confer only a small portion to the total (external) phenotype. The best endophenotype candidates (to explain the external phenotype) will also be functionally associated with aspects of the external phenotype.

## 3. From Genotype to Phenotype in Livestock Science

During the last few decades, phenotypes of livestock have been the subject of intensive genetic and genomic-based selection, often resulting in marked improvement of the trait [28]; for example, increased milk yield in dairy cattle, high egg laying capacity in chicken and high body growth rate and carcass dressing percentage in meat producing broilers, cattle and pigs [28,29,30]. Most phenotypes important to livestock production relate to complex traits because many genes interact to produce milk, eggs and muscle tissue. Complex traits in livestock are regulated by the combined action of the animal’s genotype and the effect of numerous environmental factors, i.e., how the genotype is translated into an external phenotype. The genotype of an animal sets the limits of the phenotypic variation, but the environmental factors also largely determine the expression of the genome via interaction with the genome. It has already for a long time been recognized that a phenotype (P) is the genotype (G) + the environment (E) + the interaction between genotype and environment (G × E) (P = G + E + G × E). This is true for all quantitative (complex) traits. Thus, genotype and environment interact to determine the complex external phenotypes of livestock.

**Figure 1:**
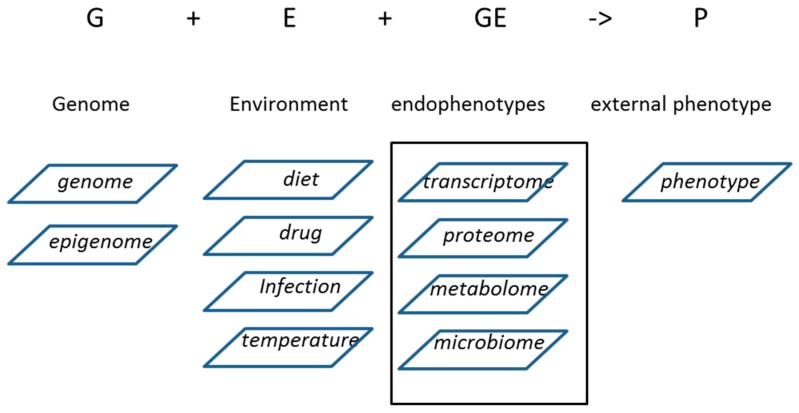
Genotype and environment affect the endophenotypes of an animal, and together, they regulate the external phenotype, or trait.

Exploring the genotype-phenotype relationship of livestock to explain the complex traits in livestock science requires investigating the genome (genomic and epigenomic variation), genome expression (transcriptome and proteome) and the genome functioning (metabolome) of the genes in relation to the pathways and networks that the genes are active in (Figure 1). It should be noted that these biological levels are not independent standalone units. Rather, many feedback loops within and between the biological levels ensure tight regulation of the expression of the genome and thereby of the complex traits. Next, we can use that knowledge for modulation of the expression of the trait, e.g., for breeding organization, to help in the identification of relevant single nucleotide polymorphisms (SNP) and susceptibility to increased/decreased environmental regulation of phenotypes. Additionally, the relation with the microbiomes, especially the microbiome of the gut, is required: there is a direct contribution of livestock-associated microbiomes to quantitative traits [31,32]. Below, we will first shortly review the individual biological levels before discussing the options and expectations of integrating them.

### Genotype

At present, the genomes of most livestock species have been sequenced [33,34,35]. Due to declining costs of genome re-sequencing, projects like “the thousand bull project” [36], and similar in other species, have constructed extended genomic variation maps [37,38]. Despite a history of decades of genetic selection, the genomes of livestock species still show a high degree of variability, including SNP, insertions/deletions and copy number variations. After decades of selection, the number of SNPs was reported to be approximately 11–17 million in cattle populations [39,40], or one SNP per 443 bp [41,42], 6.6–14 million in pig populations [43], or one SNP per 609 bp [44], and approximately three million SNPs, or five per 1000 bp, in chicken populations [45]. In humans, this figure is about one SNP per 1–2 kb [46]. In genome-wide association studies (GWAS), this SNP variation is compared with the external phenotypes of livestock to evaluate the genetic background of traits. Usually, GWAS only explain a part of the phenotypic variation, suggesting that more factors must play a role in the expression of the trait, which most probably is the influence of the environment on the expression of the genotype. In GWAS, variation in the genome that affect the phenotype are highlighted on the chromosome as QTL [47]. For many livestock species, QTL maps are constructed showing the many genomic locations related to complex traits [48]. Interestingly, many QTL are located outside annotated coding genes, emphasizing the importance of understanding variation beyond the coding part of the genome. For example, there are indications that important QTL are located in regulatory elements. As expected for complex traits, each trait is associated with multiple loci spread over many chromosomes. However, despite all present knowledge on the primary structure of genomes, the causal nature of most mutations affecting complex traits remains elusive [49].

The present status of genotype determination and knowledge of the phenotypic effects linked to genomic variation, measured with tens-of-thousand, up to a hundred thousand SNPs, enables one to use this genotype information in breeding through so-called “genomic selection/prediction” [50,51,52,53]. In genomic prediction, the relation between genome-wide genomic variation and important commercial traits within a breeding reference population is determined first and then used to select animals for the breeding value of the commercial trait without any information on the phenotype that they or their offspring will express. Use of pre-selected animals in a breeding program improved genetic progress by reducing the generation interval and decreased the cost of the breeding program. However, it should be noted that the association between genetic markers and traits should be repeatedly established after a number of generations [54,55,56]. At present, we are only at the start of these developments. In the future, the genomic information may be used to pre-select animals for a specific mating program (genomic mating) or pre-select animals for a specific environment (farm, animal management, feed composition), etc.

## 4. Functional Genome

It is important to realize that many (differences in) phenotypes are caused by differences in gene expression rather than by variation in the coding sequences of the genes [57]. Variation in gene expression is regulated by a complex regulatory network called the functional genome. The functional genome consist of a number of different (epigenetic) regulatory components, including regulatory DNA sequences, DNA methylation, histone modifications and regulatory non-coding RNAs (ncRNA), together acting in a complex network regulating gene expression, which in turn determines the so-called expressed QTL (eQTL). Unfortunately, while the annotation of the functional genome in humans is developing [58,59,60], the annotation of the functional genome is still limited for livestock animals. An ongoing effort (The FAANG (Functional Annotation of Animal Genomes) project) [61], aims to improve the annotation of the functional genome for livestock. The current status of the annotation of the functional genome probably is only a snapshot of it. The lack of good annotated functional genomes hampers the understanding of GWAS results and their use in “weighted” (i.e., using biological knowledge) genome prediction. Furthermore, it will facilitate the understanding and meaning of the biological processes underlying the complex (endo) phenotypes.

### 4.1. Epigenome

Apart from differences in the genetic code of the DNA, the expression of the genes in the genome is also regulated by chemical modifications of the bases of the DNA (e.g., methylation) and of the histones. It is a combination of all of these factors (genetics and epigenetics) that determine expression. Epigenetics modifications can change during life and can also be tissue-specific or even cell-specific. The genomic DNA can be methylated and de-methylated, and especially, the level of 5-methyl cytosine of CpG sites in so-called CpG islands in the promoter area plays important roles in gene regulation (i.e., low methylation promotes gene expression) [62]: methylation is strongly associated with gene silencing in a variety of biological contexts, and it is generally believed that the global genome methylation is to prevent repetitive element expression/expansion [63]. Different types of epigenome modifications exist: heritable vs. non-heritable, paternal/maternal, age specific, tissue specific and environmentally induced [18]. Especially, developmental processes are regulated via methylation of DNA [64,65]. However, also late in life, gene silencing via these mechanisms occurs, e.g., parent-specific gene silencing, like the *IGF-2* gene in pigs, is important for the regulation of complex traits [66,67]. Orozco et al. [18] showed that 85% of the associations related to methylation were not identified using traditional GWAS. These authors showed that in mice, natural variation in methylation levels contributes to the etiology of complex (clinical) traits.

Similarly, chemical modification of histones can regulate the access of transcription factors to the DNA [68]. Histone methylation occurs at a number of distinct lysine residues. For example, H3 can be mono-, di- or tri-methylated at lysines 4, 9, 27 and 79. Contrarily, in general, histone acetylation is a transient modification associated with active promoters and enhancers [69,70]. The combination of DNA methylation and the histone modification markers affects the affinity towards chromatin for numerous proteins [71]. As a part of the gene expression regulatory process, the ENCODE (Encyclopedia of DNA Elements) project (see above; [57,58,59]) aims to identify all functional (including regulatory) elements in the human genome (reviewed in [71]).

Importantly, from a breeding point of view, many traits appear to be regulated in an imprinted way. The underlying biological regulatory mechanism of imprinting relates to epigenetic/epigenomic modifications, which are partly inherited and partly influenced by the environment. Experiences caused by the environment may leave an epigenetic mark in the genome that may be inherited. If the experience is in early life, it may affect performance late in life. Orozco et al. [18] showed that in mice, DNA methylation is highly variable among individuals, and only 7% of methylated CpG sequences in the genome were regulated by genetic variation. Furthermore, they showed that DNA methylation is associated with complex metabolic and molecular traits, indicating the importance of epigenetics for complex traits. This again points to the importance of gene expression levels for complex traits, which will be reported in the next section.

Environmental regulation of the epigenomic processes is also important, since it may be lasting over several generations. One important example of this relates to the Dutch hunger winter. During the last winter of the Second World War, pregnant women were underfed. At present, major effects of this have been shown in the third generation on traits like insulin resistance [72,73,74]. Apparently their genomes are still epigenetically programmed to be very efficient in the use of nutrients. Thus, when studying the environmental effects on epigenetics-regulated traits, the environmental effects of pre-ancestors should also be kept in mind.

As an example: an importantly-changing environmental factor may be global temperature. Global warming changes lead to a more variable environment, and this will require more robust animals for different temperatures. The temperature of the environment may affect the developmental processes in the egg of chicken, but also regulate the temperature resistance of the chicken in later life [75]. This points to the importance of the effects of early environmental clues during the entire life time. At present, there are no examples of the application of epigenome knowledge in livestock. However, expectations for the future are high, especially in breeding and nutrigenomics. Furthermore, human studies have shown that epigenetic markers are good predictors of future phenotypic outcomes [76,77]. Thus, by understanding the contribution of epigenetic variation to phenotypic variation, a better prediction of an individual animals’ adaptation to specific and/or variable environments is expected. For example, adaptation to global warming will be necessary for all species. Since time probably is too short to select for genetic adaptations (or de novo mutations) for global warming, it could be that epigenetic adaptations are feasible due to their link to/regulation by the environment. Thus, knowing which breeds or individuals have the highest capacity to adapt to a changing environmental conditions will be beneficial for the industry. Technically, epigenetic modifications can be measured. The main bottlenecks at this moment are a lack of knowledge of the regulation of epigenetic modifications, the high costs of laboratory measurements methylation), technical challenges (histone modification on tissues) and collecting the right tissue at the correct time of development. It is known that epigenetic modifications of the genome differ between tissues and even between cells in a tissue [78]. Thus, there is a heterogeneity of epigenetic modifications for each trait. Therefore, the road to efficient commercial application of epigenetic modification is still long. However, due to the importance of the issue, it is a road we have to take quickly.

### 4.2. Expression: Transcriptome and Proteome

The level of expression of the genome and the dynamics in time and location regulate many complex traits in livestock [57]. In the previous sections, we have seen the importance of the genotype and environmentally-induced epigenetic modifications for the transcriptomic activity of genes in the genome. Apart from this, environmental factors can also directly influence the transcription of the genome. For example, many nutrients in food are sensed by the cells and regulate the expression of the genome of these cells in response [79]. One example is the mTOR signaling system. The mTOR signaling pathway integrates both intracellular and extracellular signals, especially with regard to nutrient availability, and serves as a central regulator of cell metabolism, growth, proliferation, immune signaling and cellular survival [80,81].

The regulation of transcription is a complex process in itself. Transcription initiation requires the construction of a protein complex at the promoter site of a gene, including general and gene-specific transcription factors, which can be regulated by the genotype and the epigenomic modifications of the genome [82]. For protein coding genes, transcription has to be followed by translation, which is controlled by (1) post-transcriptional regulation of the mRNA [83,84]; (2) binding of non-coding RNAs (ncRNAs) [83,85] and (3) ribosomal efficiency processes [86]. Especially, the role of ncRNAs in gene regulation has received much attention recently. Several ncRNA classes ranging from long non-coding RNA to microRNA were found, and especially, the list of identified microRNAs (miRNAs) has grown and is still growing rapidly [87,88]. The major function of miRNAs is to bind to mRNA to regulate (prevent) its translation. A good example of the miRNA effect on a livestock trait is double muscling in the Texel sheep breed [89]. Contrary to the double muscle trait in cattle, no mutation in the coding sequence of the *myostatin* gene was found. Instead, a mutation in the mRNA outside the coding sequence created a binding site for a miRNA, thereby blocking translation [89]. Furthermore, the number of annotated lncRNAs (long non-coding RNAs) has grown: there are now more annotated lncRNA genes in the human genome than annotated coding genes. However, for most lncRNAs, the functional relevance still needs to be determined. Finally, for transcriptome expression levels, the RNA degradation should be mentioned [90]. The rate and amount of RNA degradation regulates the expression level as much as the RNA synthesis does.

The transcriptome equivalent of the genomic QTL is the expressed QTL (eQTL). For eQTL, variation in the expression profiles of genes is the determining criteria. Variation in the eQTL related to phenotypic differences has been shown for numerous traits during the last few decades. However, one should remember that this variation relates to the genotype combined with the effect of the environment. Therefore, application of eQTL in commercial breeding is not similar to the use of genomic QTL in genomic selection (for a discussion, see [91]). The eQTL are used to develop biomarkers to monitor, diagnose and predict phenotypes (see below). While it is possible to use eQTL for the selection of animals [92], at present, to the best of our knowledge, this is not practiced yet by breeding companies. The main reason for this may be that the structure of the breeding schemes is not optimized for using this type of data, together with the unfamiliarity of the breeding companies with the potential of the eQTL and unavailability of data in the breeding lines. In human medicine, the use of blood transcriptomes for predicting the risk of diseases shows the potential of using eQTL [93]. The method also offers great potential for combining QTL and eQTL [94]. This offers the potential to increase the genetic progress rate further.

While protein expression level is important (e.g., for the structural protein regulating the shape and functionality of the cells), the regulation of protein activity via post-translational modifications for metabolism is another aspect of the functional genome. Phosphorylation of proteins is an important biological mechanism regulating the activity of the proteins. Since proteins act in pathways or networks, the coordinated expression and activation of proteins are a serious issue to consider (see below) [95]. Finally, protein degradation retracts proteins from the activity level. Protein degradation removes non-functional and damaged proteins, but it may also be a protein activity regulatory mechanism [96,97]. The fact that several different protein degradation mechanisms exist may point in this direction.

The environment influences the proteome at various levels; first of all, via the biological synthesis levels: the genome, the epigenome, the transcriptome and the proteome levels. As discussed before, all of these levels are influenced by the environment, which affects their expression level. However, once proteins are expressed, there are often a number of activity regulatory steps activating or deactivating the functionality of the protein, e.g., via phosphorylation, the availability of cofactors or the association with other proteins to form active complexes (complement). The environment can influence the cascade of enzymes to phosphorylate or dephosphorylate the proteins, thereby regulating the proteome functionality.

### 4.3. Biological Function: Metabolome

One could argue that biological functioning largely involves metabolism for many traits, although other traits are regulated by proteins (e.g., immunology). Many non-structural cytoplasmic proteins are enzymes facilitating chemical reactions with substrates. Since the product of one reaction becomes the substrate for the next reaction, many (intermediary) metabolites exist. While the number of genes, mRNA and proteins in livestock are reasonably well known, the number of metabolites remains to be established, but estimates from the human metabolome project suggest that at least half a million metabolites exist, of which, roughly 70% is known at this moment [95,96,97,98,99,100,101,102]. Many metabolites originate from the feed, the gut microbiota and a series of degradation/synthesis reactions in the host that generate the high variety of metabolites. Some metabolite reactions are important for all cells, including energy metabolism. However, since organs and tissues have distinct functions, different organs and tissues generate/use different metabolite profiles. Notable to mention is that the microbiome also produces quite a few (essential) metabolites (for more information about the effect of the microbiome, see below).

Some complex traits may directly relate to the metabolite profile, for example the metabolites generated in energy metabolism. Many performance traits (e.g., growth rate, feed efficiency, etc.) relate to this metabolism. For example, the combination of metabolites in meat and adipose tissue may directly constitute the sensory meat quality traits of taste and smell [103,104,105,106]. Other traits may be a combination of structural protein composition and metabolites, like palatability. The lack of knowledge about the existing metabolites in the tissues and how to measure them hampers the knowledge about the influence of the metabolome on complex traits, but it is clear that many complex traits are influenced/determined by the metabolome, often requiring the interaction of several/many reactions to create the best combination of metabolites for the trait.

The metabolome is also influenced by the environment. Firstly because the metabolome depends on enzymatic reactions of the host and the microbiome for which the environmental influence on the proteome (enzymes) is important (see above), but also because metabolites taken up from the feed can differ because of differential feed composition.

### 4.4. Networks and Pathways

In biology, genes, proteins and metabolites are not stand-alone units. Proteins, for example, interact with each other either directly, i.e., to form complexes of peptides functioning together, or indirectly, i.e., via the reactions of the metabolites (see above). Such interactions can be described in pathways and networks [95]. Pathways often describe known physiological interactions in specific tissues, many of which can be found in databases [107,108,109]. Networks of genes, proteins or pathways usually are visualized by specific software, such as Cytoscape [110], or on the Internet with STRING (Search Tool for the Retrieval of Interacting Genes / Proteins) [111] or STITCH (Search Tool for Interaction Networks if Chemicals and proteins) [112], and several software packages written in R (see Bioconductor [113]) enable the creation of multi-datatype networks merging biological information on several biological levels. It is important to note that these software tools are very different, e.g., while Cytoscape is a data visualization tool, STRING and STITCH analyze the data using public domain data; STITCH adds available metabolite data to the analysis. Therefore, using these tools together in the analysis of data provides more insight than using just one of these tools. Since the number of tools in Bioconductor continuous to grow, we do not discuss the present situation: the interested reader is encouraged to explore the site for the specific interest.

Although networks are generated based on known interactions between proteins, specific information about tissue functionality is often lacking. Furthermore, the available knowledge is often generated in humans and laboratory species (mice, rats, yeast) and is rarely available in the livestock species. Further bioinformatic analyses may provide information about known biological functionality in a specific tissue.

Pathways and networks may also explain the concept of epistasis (what can be defined as “the phenomenon that the effect of one gene is dependent on the presence of another”): the same phenotype can be caused by different genes. Genes may cooperate in the same pathway or network; thus, although the genes are different, through participation in the same pathway/network, the underlying biological mechanism and its outcome, the phenotype, may be the same. Thus, breeds may differ in QTL (genes for the same trait), but share a biological mechanism developing an external phenotype. Thus, genotype-phenotype relationships should include this [114].

To make it even more complex, the flux through a pathway does not equally relate to all proteins, and control of the output of a pathway is often done by a (number of) rate-limiting protein(s), e.g., an enzyme [115]. Regulation of such proteins us in generally more important than regulation of other proteins in the same pathway. Furthermore, pathways and networks may be put together in a higher order network of pathways or network of networks [116]. Such a higher order of biological information often is very complex to understand because it brings together large amounts of data. Therefore, systems biology approaches can be used to describe these complex arrays of information in (mathematical) models [117,118,119]. These models have to be accompanied by knowledge of the interactions among the different biological levels of the endophenome (see above). Together, they model the biology of complex traits, hereby facilitating a better understanding of the trait, which may be used to develop “predictive biology” for the trait. In the discussion below, we will further elaborate on this.

**Figure 2:**
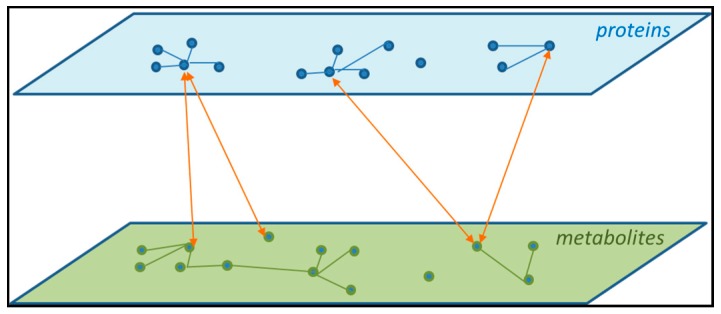
Interactions among members within and between two biological levels, protein and metabolites.

Figure 2 shows an example of two layers of biological information: proteins and metabolites. The figure shows that there are interactions among members within a layer. Within the protein layer, hub proteins showing connections with many other proteins are central in a network. Furthermore, there are direct connections between the two layers. This shows why a (mathematical) model describing the endophenotypes requires one to include all biological levels and include the interactions among them.

### 4.5. The Influence of the Microbiome

On the skin and all body cavities of animals, a microbiome is living that can affect many aspects of the life of the animal and (co)determine phenotypic traits. In recent years, the gut microbiome has received much attention because it has been recognized that this microbiome is important for (1) the health of the animal; (2) the metabolism of the animal; and (3) the development of tissues and organs of the body of the animal [120,121,122,123,124]. Health, metabolism and development are all complex traits highly important for livestock productivity. The gut microbiome can be regarded as part of the environment, but it also consists of a large number of genomes: the genetic potential of the microbiome is orders of magnitude greater than the genetic potential of its host. Therefore, it can also be argued that the genome of the animal is associated with a variable microbiome, which is partly determined by the animal’s genome. However, it is known that the diet and also the environment greatly affect the composition, diversity and metabolic activity of microbiomes.

The gut microbiota start to develop during birth [23]. Although the diversity and composition of the gut microbiome can be stable during (part of) the animals life, several environmental factors affect the composition and diversity of the gut microbiome. The most important environmental factor is feed composition. Especially in mammals, weaning results in major changes of the composition of the gut microbiota [23]. For example, in pigs, it has been shown that the microbiota starts to develop after birth, reaching a more or less stable composition at Day 15. After weaning, major changes of the composition occur, but reaching a second stable composition at about five weeks of age. If undisturbed, this period can last until slaughter [23]. Other factors influencing the stability of the gut microbiota include stress [125,126], infection, use of antibiotics, etc., but also, to a lesser extent, the genotype of the animal [127,128]. This further illustrates the importance of the interaction between the genotype and the environment of the animal.

The gut microbiome is also an important metabolic “organ” interacting with the metabolism of the host [129]. Several of the metabolites synthesized by the gut microbiome are taken up by the animal and metabolized further in specific organs and tissues body wide. Recent evidence shows effects of gut microbiome-derived metabolites on animal health, performance traits, like growth and feed efficiency, and even the brain [124]. These studies clearly show a direct effect of the gut microbiome on complex traits of livestock. It can be argued that the gut microbiome constitutes a part of the metabolic capacity of the animal. Since cells can sense many food-derived metabolites, there may be a direct interaction between the metabolomes of the microbiome and the animal. We will discuss this latter together with the information from other biological regulatory levels reviewed above.

## 5. The Biology of Complex Traits

We have discussed that the biology underlying complex traits in livestock relates to the genotype and regulatory sequences of multiple genes, the effects of the environment resulting in modulation of the epigenome, the expression of the genes via diverse transcriptional mechanisms and post-transcriptional expression processes modulating the activity of the proteins and subsequently the metabolome. Two important questions remain: (1) how can we integrate all of this knowledge to explain the regulation of the complex traits; and (2) how can we use this knowledge to modulate the complex traits? The answer may be that we need to start thinking of biological systems instead of a collection of genes or components. Therefore, we need to do experiments using a systems-driven research and perhaps, in the future, change from “genomics prediction” to “systems (biology) prediction”.

### 5.1. Integration to Explain the Regulation of Complex Traits

Integration of data derived from different biological levels is one of the main objectives of the system biology discipline [118,119]. Software tools exist to start such work. They typically require measuring all levels of data on the same individual. Since the current available data for such analysis usually originate from several experiments, it is still needed to collect such data on material derived from the same animals, but this is going to be timely, labor intensive and very costly and, therefore, probably only possible for important reference populations. However, the analysis of such data in commercial breeding populations is needed to study the complex traits in these populations to improve the genetic potential of the animals; and therefore, it is recommended that in future experimental design in livestock science, the use of systems-driven hypotheses will be central. Because of the influence of the interaction between the genotype and the environment on the phenotype of complex traits, it is required to study these traits in the animals expressing such traits in their own specific environment. Samples should be taken from the same animals, preferably at the same time points, and all analyses should be done independently on these samples before integration can start. Above all, phenotypic measurements should be done on these animals, and samples should be stored for measurements of new phenotypes when available. Phenomics is a new omics discipline. New software tools are developed generating new insights for phenotypes. These tools also indicate what and how to measure the phenome and how to integrate these data with other omics data. For example, Hiller et al. [130] used a forward genomics approach to link the genotype to the phenotype. The method encompasses genome-wide screens for metabolic phenotypes among related species.

A database archives human genome-phenome relationships for biomedical research [131]. Brookes and Robinson [132] reviewed a large number of specific human genotype-phenotype databases especially dedicated to (specific) diseases. Here, the interested reader also finds links to these databases. They also developed a software tool focusing on discovering genes based on user-specific disease/phenotype terms [133,134]: (quote) Phenolyzer includes multiple components: (i) a tool to map user-supplied phenotypes to related diseases; (ii) a resource that integrates existing knowledge on known disease genes; (iii) an algorithm to predict previously unknown disease genes; (iv) a machine learning model that integrates multiple features to score and prioritize all candidate genes; and (v) a network visualization tool to examine gene-gene and gene-disease relationships. Several similar software tools exist. They all investigate the relationship between (disease) phenotypes and genes, but the intermediate layers, which we show to be of major importance, are less or not investigated with these tools. In addition, Shah [135] describes efforts to develop the Phenome Knowledge Base [136], a database aiming at making the phenome data available and analyzable. Often data from multiple sources are difficult to combine. However, efforts are ongoing to make high-throughput data from multiple sources analyzable by integrative genomics efforts [137].

Zhang et al. [138] developed a simulation tool for pedigree, phenotypes and genomic data. It includes analyses of population, trait and genetic architectures data, and it can simulate multiple genetically-correlated traits with desired genetic parameters and underlying genetic architectures. Pathway-based methods group mutations from genes in a biologically-relevant pathway [139]. Phen-Gen is a method that combines the phenotype and the genotype aiming to analyze rare traits [140]. The method includes an online software tool to analyze data [141]. Rare traits may be the result of single cell differential phenotypes. RaceID is an algorithm that identifies rare cell types in complex populations of single cells [142]. The overall message of this paragraph is that there are a number of tools available, and the number is still growing; but at present, these tools are all unrelated and each with different functionality.

It is clear that at present, human (disease study) and mouse (traits of inbred strains) are used to develop such tools [143]. Although these tools are not used in livestock science yet, they promise high value for future data collection and analysis of complex traits in livestock.

Above, we discussed the external phenotype and the endophenotypes. While external phenotypes in general differ in time and body localization, endophenotypes are much more dynamic. There are several reasons for this dynamics: (1) even small environmental changes may affect the expression levels of genes, proteins and metabolites; (2) some phenotypes relate to biological processes taking place earlier than the measurement of the phenotype (e.g., muscle development takes place during life, while meat quality develops after death; programming the immune system takes place before the immune system is challenged by a pathogen, etc.); (3) expression profiles differ between cell types in a tissue, and even within a tissue, individual cells of the same cell type differ in expression profile and also vary in time [144,145]. Thus, it is a challenge to measure the right tissues or cells.

Another major challenge is sample size. Sample size determines the power of an experiment and, thus, the ability to draw conclusions from the results. For all omics technologies, required sample size depends on a number of variables. For example, Orozco et al. [18] used genomics, epigenomics, transcriptomics, proteomics and metabolomics in 90 mouse lines, using only 3–4 animals per mouse line for several of the endophenotypes. From a livestock perspective, these numbers may appear to be low, but it should be noted that here, highly inbred mouse lines were used. Livestock species typically have a more outbred character, which may require higher numbers of animals to reach the same effective sample size. The generally used rule of thumb in genomic selection is to use a reference population of at least 1000 animals [52]. Current applications of genomic selection in breeding programs only involve the use of genomics, but not any of the other omics layers. In general, when the number of animals (*n*) is very small compared to the number of parameters (*p*; i.e., SNPs, or gene-, protein- or metabolite expressions), the regular statistics cannot be used. This problem, commonly known as the *n* << *p* problem, has been solved in methods applied for genomic selection [146]. What remains is the question of how to determine the required number of animals needed within the experiment, depending on the objective and which omics technologies are involved. Suravajhala et al. [147] stated: The advantage of performing GWAS in livestock species over humans is the availability of related animals and subsequent knowledge about the pedigree, which greatly reduces the number of individuals needed to reach sufficient power to detect genetic variants associated with the trait of interest (quote). Measuring external phenotypes (i.e., traits) is regularly done on large numbers of animals in breeding programs. This suggests that the number of animals required for the task is not as large as considered in humans. In fact, Vazquez et al. [148] managed to show that using combined omics technologies improved the prediction accuracy of survival of breast cancer in humans, when using less than 300 samples. This seems to confirm the power of experiments using a reasonable number of animals. However, at this moment, there is no final answer to the question how many animals are needed for such an experiment. Knowing that it is feasible to investigate the endophenotypes, knowing that the genotype and the epigenome are (partly) heritable and knowing that the environment in the barns where commercial livestock animals are held is relatively stable (as compared with the outside environment), we argue that it is important to investigate the endophenotypes of complex traits in livestock. Since the endophenotypes consist of the heritable parts and the environment, it is possible to make predictions for the next generation enabling one to use the endophenotypes in breeding. The collection, storage and handling of such large amounts of data poses specific problems. Recent “big data” projects are developing tools for this. Feltus et al. [149] recognizes that the combination of biologists, mathematicians and IT specialists is often an arduous relation. Therefore, they review methods to transfer big data across networks, especially for biologists in order to frame their genomics-oriented needs to enterprise IT professionals. Specifically, they discuss four key areas: (1) data transfer networks, protocols and applications; (2) data transfer security including encryption, access, firewalls and the Science DMZ (a network design pattern for data intensive science); (3) data flow control with software-defined networking; and (4) data storage, staging, archiving and access (quote). Since data derived from different disciplines are merged together, a good ontology may be needed [150]. These steps make the data available for large-scale analyses, such as described by [151], using parallel computing. They describe and present links to many software tools that can be used (which is not the aim of the present paper). These authors point to the numerous tools in Bioconductor for this aim (see the discussion above). For instance, cloud computing is an interesting strategy emerging as a solution applied in several bioinformatics areas. Tavaxy, which can also be used in the cloud [152,153], Pegasus [154], Swift/T [155] and SciCumulus [156] are some examples of scientific workflow systems that are able to manage bioinformatics experiments in cloud infrastructures. 

Wanichthanarak et al. [157] reviewed the strategies to integrate genomic, proteomic and metabolomic data. Big datasets may result from such integration. To name a few: these authors specifically mention methods, such as IMPALA (Integrated Molecular Pathway Level Analysis) [158], iPEAP (Integrative Pathway Enrichment Analysis Platform) [159], MetaboAnalyst (processing, analyzing, and interpreting metabolomic data) [160], SAMNetWeb (Simultaneous Analysis of Multiple Networks) [161], etc. Importantly, there is also a focus on integration via pathways and network biology analysis. The number of practical applications of this in livestock science is still scarce. 

While software tools are important, we should not wait until all of these tools have been developed. Examples in reference animals such as mice show the possibilities at this stage. Excellent examples are the studies of Orozco et al. [18] and Benis et al. [162], both using mice as sources of experimental data. In a highly interesting study, Orozco et al. [18] showed how the genotype affected some part of the methylome, while other parts were environmentally affected, how the profiles of gene expression were regulated at the RNA and proteome levels and how the metabolome profile reacted. Benis et al. [162] showed the integration of five biological levels: microbiota, transcriptomics, cytokine and metabolomics in serum and urine, pointing towards several connections between the layers and within the layers. This clearly shows the power of such studies for understanding complex traits by linking the genotype and several phenome levels to the external complex trait phenotype. 

**Figure 3:**
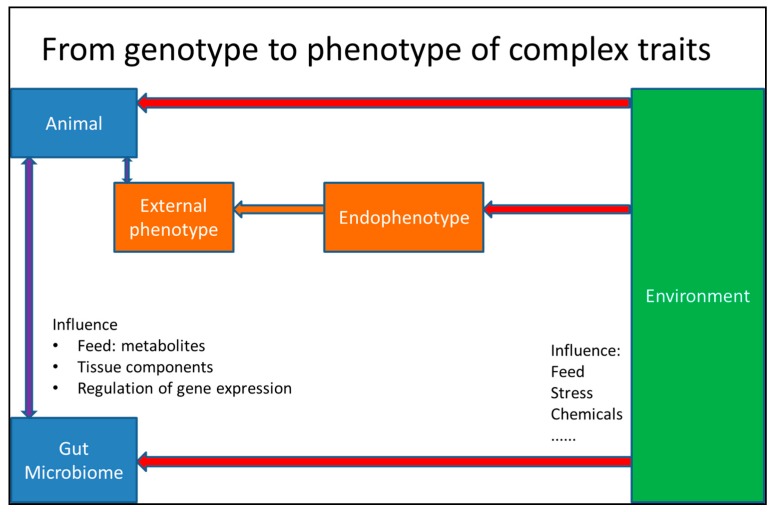
The interactions between the environment, the animal and the gut microbiome; and the direct interactions between the gut microbiome and the animal. The blue boxes represents the genome(s) of the animal. The orange boxes represent the phenotypes of the animal. The green box represents the environment. Red arrows indicate (in) direct effects of the environment. Purple arrows indicate two-way interactions. It should be noted that it is possible to regard the composition and diversity of the microbiome as an endophenotype (in which case, the box should be mixed blue/orange). If we consider this, an additional red arrow from the microbiome box to the endophenotype box should be added.

Although integrating less biological levels, studies on human complex diseases, such as metabolic syndrome, also provide good examples on this [163]. The interaction between genome and environment to regulate complex traits was clearly shown. By integrating the results of the composition of the gut microbiome with the body-wide gene expression and metabolome effects, a highly integrated multi-genome, multi-gene, interactive picture of the regulation of complex traits arises (Figure 3). It shows how potential interactions may determine variations in traits and what to study if such trait variation were useful for improvement for health or the productivity of livestock.

### 5.2. Improve Complex Traits

Meanwhile, can we do nothing to improve complex traits in livestock before having collected and analyzed all of the data mentioned above? Although the above-mentioned information is needed to explain the regulation of a complex trait and this information is needed to guide the improvement of a complex trait, partial information associated with a complex trait is still useful to improve the phenotype of a complex trait. It should be mentioned here that association is a statistical relationship, thus not necessarily a causal relationship. However, if we remind that genes work in pathways and proteins interact in networks, then we can rely on groups of genes or proteins (and/or related metabolites) that relate to the same pathways or networks indicating the same biological mechanisms [164]. Such genes, proteins or metabolites are called biomarkers [165,166,167,168,169]. Biomarkers may be single genes, proteins or metabolites, or may be composed of several of them, or even combinations of them.

To understand the concept of biomarkers, knowledge of the biological pathways highlights potential genes (proteins, metabolites) that can act to monitor the activity of the pathways, which relate to the establishment of the phenotype of the relevant traits. Knowledge of the relation between the value of the biomarker and the phenotype of the trait can be used to predict the trait by only measuring the value of the biomarker. The biomarker profile may be used as an indicator (marker) for the endophenotype (similar to eQTL and external phenotype): variations in genotypes may be linked to endophenotypes. In turn, endophenotypes may be linked (functionally, statistically) to external phenotypes. Biomarkers may be especially important for traits that are difficult to measure or where measuring is expensive. For example, meat quality traits can be measured only one to several days after slaughtering or for certain products (e.g., dry cured ham) even months or years post mortem. Since such products are very expensive, starting with high quality meat is important. Biomarkers can be measured directly after slaughtering and can be helpful to predict the quality of the ham months (or even years) later.

Furthermore, during an intervention study (e.g., change of feed composition), the outcome of the intervention may be predicted before the experiment starts and monitored during the intervention. Biomarkers may also be used as diagnostic tools in livestock; for example, to study the interactions between phenotypes if traits share common biological pathways [167,169].

Developing biomarkers in a population establishes the relation between the value of a biomarker and the phenotype of a trait in that population. Because of lacking information (as discussed above), the general biological mechanisms underlying the complex trait remain elusive, and the biomarker relates to that specific population. However, validation in an unrelated population, preferably a different breed, is required [170]. If the same relation between biomarker and phenotype of the complex trait has been found in two independent and genetically-different populations, it strengthens the biomarker, although still, no causal relation can be concluded [171,172,173]. Therefore, it remains possible that in future generations, different biomarker effects may be found or that the association may be lost. This is similar to the use of genome-wide genotypes in genomic breeding, where the association between SNP profiles and complex traits is established in a population and used to preselect breeders for a test phase or even to select breeding animals directly. It may be necessary to re-establish the association regularly. However, if genetic markers and biomarkers can be used together [174] and combined with additional data, such as gut metabolome profiles, a comprehensive analysis will be possible, leading to real understanding of complex traits and establishing causal testing methods to improve complex traits of livestock [175].

Summarizing, we have described a road leading from the genotype and the environmental effects via endophenotypes towards understanding of the biological regulation of traits. We discussed the importance of the integration of biological knowledge from all biological “levels”. Using all of this information, we discussed a road to implement the knowledge for improvement of complex traits in livestock science.
